# Role of Stressful Life Events, Avoidant Coping Styles, and Neuroticism in Online Game Addiction among College Students: A Moderated Mediation Model

**DOI:** 10.3389/fpsyg.2016.01794

**Published:** 2016-11-22

**Authors:** Huanhuan Li, Yingmin Zou, Jiaqi Wang, Xuelin Yang

**Affiliations:** ^1^Department of Psychology, Renmin University of ChinaBeijing, China; ^2^Department of Psychology, Sun Yat-sen University, GuangzhouChina

**Keywords:** online game addiction, stressful life event, coping strategies, neuroticism, moderation analysis, mediation analysis

## Abstract

Online game addiction (OGA) is becoming a significant problem worldwide. The aim of this study was to explore the incidence of OGA and the roles of stressful life events, avoidant coping styles (ACSs), and neuroticism in OGA. A total of 651 Chinese college students were selected by random cluster sampling. Subjects completed the Chinese version of Young’s eight-item Internet Addiction Scale (CIAS), Online Game Cognition Addiction Scale (OGCAS), Revised Eysenck Personality Questionnaire Short Scale in Chinese (EPQ-RSC), Chinese College-student Stress Questionnaire, and Coping Style Questionnaire. Structural equation modeling (SEM) was used to explore the interactive effects of stressful life events, ACSs, and neuroticism on OGA. Of the 651 participants in the sample, 31 (4.8%) were identified as addicts. The incidence of OGA was two times higher for males than females. The addicts had markedly higher scores on the neuroticism subscale of the EPQ-RSC than non-addicts. Compared to non-addicts, addicts were more apt to use ACSs. Having an avoidant coping strategy mediated the effect of stressful life events on OGA. Furthermore, neuroticism moderated the indirect effect of stressful life events on OGA via ACSs. Applications of these findings to etiological research and clinical treatment programs are discussed.

## Introduction

Online game playing has become a major leisure activity among young adults, particularly in China, where some 13 to 15 million 18–24-year-olds have been reported to engage in so-called persistent-world games (Hosoi, 2005), with nearly half of this population being college students (China Online Game Users Survey Report, 2008). Jiang et al. (2007) found that 55.9% of college students reported playing online games during their leisure time, and 67.5% of these students were male.

The allure of online gaming among young adults may be attributable to their engaging, well-developed immersive environments and their providing a medium of interaction among large numbers of users, which can facilitate the development of interpersonal relationships within imaginary game worlds without any space or time limits (Yao et al., 2014). Furthermore, continued involvement was reinforced in that good game skills enhanced the player’s reputation, earning him or her respect from other players, and potentially, fulfilling a need for self-realization (Lafrenière et al., 2009).

There has been some controversy surrounding the benefits and drawbacks of online gaming. Some researchers have claimed that online games can contribute to adolescent development of intrinsic motivation by creating interactive learning environments and by fostering competition, control, collaboration, challenge, and achievement (Dickey, 2007). Others have criticized online gaming for leading to laziness and aggressive behaviors (Karakus et al., 2008). Playing videogames, defined as hedonic informational technology, which use informational technologies for fun rather than asking for help with homework can facilitate adolescent’s disengagement from school. The higher the extent of use of videogame playing, the more the adolescence disengaged from school (Qahri-Saremi and Turel, 2016). One aspect of online gaming that is clearly detrimental was online game addiction (OGA), defined as “excessive and compulsive use of computer or video games that result[s] in social and/or emotional problems; [and] despite these problems, the gamer [is] unable to control this excessive use” (Lemmens et al., 2009, p. 78).

Excessive online game playing among college students has been shown to lead to depression, anxiety, loss of appetite, sleep disturbances, and reduced physical activity (Yang et al., 2005; Kim et al., 2008). Difficulties with time management and interpersonal relationships in adolescents have also been reported to be very common due to addicts spending more time on the Internet than interacting with people in reality (Lai et al., 2013). In addition, adolescent addicts have been found to have reduced academic performance due to frequent absences from classes (Burgess et al., 2012).

Davis (2001) proposed a theoretical cognitive-behavioral model with which to explore the causes of specific types of problematic Internet use, in which an individual uses the Internet pathologically for a particular purpose, such as online game playing. According to Davis’s model, OGA is the result of a predisposed vulnerability (diathesis) and life events (stress). However, the relationship between diathesis and stressful life events in the development of OGA is unclear.

## Background

### Stressful Life Events, Avoidant Coping Styles (ACSs) and Online Game Addiction (OGA): Evidence for Meditating Model

Grounded in the cognitive-behavioral theory (Davis, 2001), our hypothesis posited that the relationship between stressful life events and OGA would be stronger for individuals with higher maladaptive cognitions or coping. College students experience stress due to academic pressure, interpersonal conflicts, job-seeking, and other factors (Ross et al., 1999; Chi and Lin, 2005). A link between stressful life events and Internet addiction in this population has been established (Zhou and Li, 2009; Yan et al., 2014), suggesting that stressors may be a robust predictor of subsequent OGA. However, coping style as a mediator has been shown to account for much of the variance in stress levels and individuals’ stress-related problems. For example, after exposure to stress, individuals who reported a greater use of emotion-focused coping (i.e., managing emotional symptoms of stress) reported poorer sleep quality than those who reported a greater use of problem-solving coping (i.e., altering stress-causing circumstances) (Morin et al., 2003). Stress in the absence of positive coping style is a very powerful predictor for substance abuse (Grusser et al., 2007). Indeed, individuals who use compulsive game-playing to cope with daily hassles or social rejection have been found to be more susceptible to addiction (Kardefelt-Winther, 2014).

It is worthy to note that classifications and diagnostic criteria for problematic Internet use diverge (Douglas et al., 2008), characteristic of this disorder subtypes and their relations to stress life events and maladaptive coping may be also different. Davis (2001) proposed two subtypes: specific pathological Internet use (SPIU) and generalized pathological Internet use (GPIU). GPIU refers to an addiction to the Internet itself and does not concern any specific online activity, while SPIU has been defined as a overuse of specific online behavior, such as online game playing, gambling, sex, and social networking. In Davis’s (2001) model, SPIU and GPIU may result from two different kinds of maladaptive cognition process. Social context of addicts, such as social isolation or lack of social support from family or friends may contribute to the cause pathway of GPIU. GPIU might be an behavioral response for coping with pressures. However, SPIU might be the result of a pre-existing psychopathology (i.e., compulsive gambling and pornography abuse). A specific online activity served as an immediate behavioral reinforcement of this psychopathology (Laconi et al., 2015). Furthermore, maladaptive cognitive styles, such as rumination, low self-worthy and self-blame are automatically enacted when individuals face with stress or interact with a stimulation associated with the Internet, which result in either SPIU or GPIU. Our previous results showed that stressful life events contributed to GPIU, and that these effects were largely mediated through ACSs, including self-blame, fantasy, withdrawal, and rationalization (Li et al., 2009). Considering that online game addicts tend to use avoidant strategies to escape everyday stressors and to avoid daily responsibilities (Kardefelt-Winther, 2014; Cheng et al., 2015), it is reasonable to postulate that stressful life events contribute to OGA mainly in individuals with ACSs.

### Moderation by Neuroticism

Among personality traits, aggressiveness and impulsivity have received major attention as predictors of OGA, whereas the role of neuroticism has been partially neglected (Charlton and Ian, 2010; Collins et al., 2012). For example, adolescents with online game overuse were more likely to have aggressive behaviors during the previous year, suggesting that aggressiveness was a relevant predictor of subsequent OGA (Ko et al., 2009b). Young adults who are characterized by high impulsivity, involvement in MMORPGs can become problematic internet user (Billieux et al., 2011).

Interestingly, findings regarding the relationship between neuroticism and OGA have been mixed. Most researchers reported that online game addicts scored higher on neuroticism (Cao and Su, 2007; Mehroof and Griffiths, 2010; Kuss et al., 2013a), but some found no differences in neuroticism between addicts and non-addicts (Wang and Gao, 2008). Understanding the reasons for these mixed results is complicated by the heterogeneous nature of the diagnostic criteria, instruments, and cultural backgrounds of the subjects. Given that drug dependence shares clinical features with OGA, and neuroticism is one of the most robust factors characterizing the drug-dependent population (Mccusker and Brown, 1991; Sigurdsso and Gudjonsso, 2009), it is important to explore the role of neuroticism, as well as other personality traits, in OGA.

Neuroticism can moderate perception of stress and its consequences (Hills and Norvell, 1991; Yu, 2013). The first way in which neuroticism may play a role in the relationship between stressful life events and OGA is by affecting for whom such a relationship is present or more pronounced. The association between Internet preference and Internet addiction was more stronger in neurotic adolescents (Lei et al., 2006; Kuss et al., 2013b). Further, neuroticism may exert a significant moderating effect in the relationship of coping styles to OGA. The notion was supported by the findings that neuroticism can predict negative coping styles, such as fantasy, rationalization, or withdrawal, especially in stressed young populations (Connor-Smith and Flachsbart, 2007). Neurotic individuals tended to be more anxious in face to face communication than Internet communication, which result in their staying online to relieve anxiety when exposed to stress (Rice and Markey, 2008). Thus, not all online game players are equally affected by stressful life events and that neuroticism may moderate the relationship between exposure to stress, ACSs, and OGA.

In summary, there is a need to explore the interaction effect of personality and coping styles on development of OGA. However, very few studies have explored interactions among neuroticism, coping strategies, stressful life events, and OGA in college students.

The aim of the present study was to examine how personality of neurotic characteristic influences the relationship between stressful life events and OGA via ACSs. Our previous works demonstrated that psychoticism and extroversion were two significant predictors for GPIU, but not neuroticism (Li et al., 2008a). The current study may contribute to PIU studies and make advances in our previous study by highlighting the different role of neuroticism in developing of GPIU and SPIU for gaming.

**FIGURE 1:**
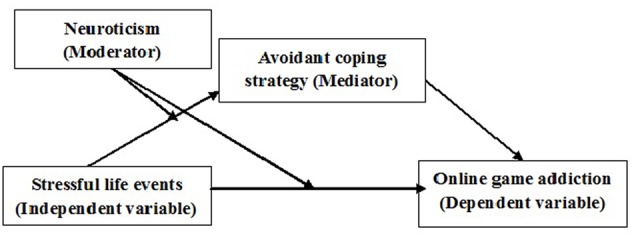
**Hypothesized mixed model**.

We propose a mixed model in which to examine mechanisms underlying the interaction of stressful life events and personality traits in predicting OGA (**Figure 1**). In this mixed model, the moderating effect of neuroticism on the relationship between stressful life events and OGA would be at least partially explained by the mediator variable of ACSs. On the other hand, an interaction between neuroticism and ACSs in determining development of OGA would suggest the presence of a moderated mediating effect.

In the present study, the incidence of OGA was determined in a sample of Chinese college students and the roles of ACSs and neuroticism on OGA were explored. We hypothesized: (1) that OGA incidence in Chinese college students, especially males, would be higher than in the general population; (2) that online game addicts would have higher levels of stressful life events, ACSs, and neuroticism than non-addicts; and (3) that the relationship between stressful life events and OGA would be mediated by ACSs, and that this mediation effect would vary with neuroticism.

## Materials and Methods

### Participants and Procedures

A random sample of three universities was selected to be surveyed by placing all universities in the Guangzhou city on a database. Random allocation was then used to select those participants in collecting the sample. Each participants was asked to complete the questionnaires.

A total of 700 college students volunteered to participate in this study for no compensation. Because 46 were excluded due to missing data or lack of Internet experience, the final sample size was 654. The cohort included 298 (45.6%) males and 356 (54.4%) females. Their average age was 20.29 years (*SD* = 1.39; range 18–22). They reported spending an average of 19.39 h/wk (*SD* = 17.12; range 1–140) using the Internet and 2.62 h/wk (*SD* = 6.32; range 0–76) playing online games. The participants were majoring in social science (economics, business administration, education, philology, law, politics, or philosophy) or natural science (math, physics, chemistry, biology, life sciences, or computer sciences). This study was carried out in accordance with the recommendations of ‘Ethic of guidelines, The Institutional Review Board of Sun Yat-sen University’ with written informed consent from all participants. The Institutional Review Board of Sun Yat-sen University approved the protocol for this study. All participants gave written informed consent in accordance with the Declaration of Helsinki. Prior to the beginning of the study, participants were informed that the purpose of the research was to examine psychological factors associated with Internet use, and assured that their anonymity and privacy would be fully protected.

### Measures

Questionnaires were administered in group format and took 20∼30 min to complete. The demographic section of the questionnaire included gender, age, major, and year in college. Participants were asked whether they played online games. Those who responded positively were asked about the duration of their playing and the average time spent playing online games each week. The full methodology has been published previously (Li et al., 2009). All the measurements used in the present study are presented in **Table 1**.

**Table 1:** All the instruments used in this study.

Scale	Item/subscale	Source	Measure
CIAS	Eight items	Zhu and Wu, 2004	Internet addiction
OGCAS	Four subscales: cognitive styles, compulsivity, withdrawal, and impaired social function related to online gaming	Li et al., 2008b	Measure of OGA
CSQ	Six subscales: problem-solving, help-seeking, self-blame, fantasy, tolerance, and rationalization	Xiao and Xu, 1996	Measure of ACSs (self-blame, fantasy, tolerance, and rationalization)
CSSQ	Five subscales: academic stress, social communication stress, job-related stress, daily hassles, and major life events	Chi and Lin, 2005	Measure of stressful life events
EPQ-RSC	Four subscales: psychoticism, extraversion, neuroticism, and social desirability	Qian et al., 2000	Measure of neuroticism and psychoticism

*CIAS, the Chinese version of the Internet Addiction Scale; OGCAS, The Chinese version of the Online Game Cognitive Addiction Scale; CSSQ, the Chinese College-student Stress Questionnaire; CSQ, the Chinese version of the Coping Style Questionnaire; EPQ-RSC, the Revised Eysenck Personality Questionnaire Short Scale for Chinese. OGA, online game addiction; ACSs, Avoidant coping styles.*

#### Internet Addiction

The IAS (Young, 1998) is an eight-item self-report scale that was used to assess an individual’s preoccupation with the Internet. A Chinese version of the Internet Addiction Scale (CIAS; Zhu and Wu, 2004) was used in the present study. Each item is scored as 0 (no) or 1 (yes). Participants fulfilling five out of eight criteria are considered problematic Internet users. Given that Pathological Gambling and Internet addiction share similar clinical features, such as craving, withdrawal, and tolerance (Block, 2008), the cut- off point of “5” was consistent with the number of criteria used for Pathological Gambling diagnosis and should be considered as a slightly more rigorous cut off score to differentiate normal from Internet addiction (Young, 1998). The five items include: repeated efforts to curtail Internet use; a need for increasingly more time online to achieve the same amount of satisfaction; depression, irritability, or mood liability when Internet use is limited; staying online longer than anticipated; and using the Internet as a means of mood regulation, typified by phrases such as “I use the Internet as a way of escaping from problems or of relieving a dysphoric mood,” “I feel the need to use the Internet with increasing amounts of time in order to achieve satisfaction.” The remaining three items include: loss of significant relationship, academic performance and career opportunity due to the Internet; lying to others to conceal the extent of indulging in the Internet; and feeling preoccupied with the Internet, typified by phrases such as “I lie to family members, therapist, or others to conceal the extent of involvement with the Internet,” “I always think about previous on-line activity or anticipate next on-line session”. The internal consistency coefficient was excellent (Cronbach’s α = 0.95) for the current sample.

#### Online Game Addiction (OGA)

The Chinese version of the Online Game Cognitive Addiction Scale (OGCAS; Li et al., 2008b) was used to examine cognitive styles, compulsivity, withdrawal, and impaired social function related to online gaming. The 16 items of the Chinese version of the OGCAS are calibrated with scores ranging from 1 (strongly disagree) to 5 (strongly agree) with higher scores reflecting a greater tendency toward OGA, typified by phrases such as “I always have a strong desire to play online game,” “Acquiring new game skills is the only way to make me happy,” “I always play online game longer than originally intended,” “I prefer to stay on the game world rather than to talk with other people in the real world,” “I feel restless, depressed, or irritable whenever attempting to cut down or stop online game playing,” “I always forget to eat or do homework because of online game playing.” In the current sample, the internal consistency of the OGCAS (Cronbach’s α = 0.95) and the four subscales (cognitive styles, Cronbach’s α = 0.71 ; compulsivity, Cronbach’s α = 0.87; withdrawal, Cronbach’s α = 0.83; impaired social function, Cronbach’s α = 0.72) was excellent.

The mean score of the OGCAS was 22.92 (*SD* = 9.22) for the current sample. Based on this data, the cutoff score of the OGCAS is set at 32, which fulfilled the criteria as a sum of mean scores and standard deviation. Two types of Internet user groups were identified in accordance with the CIAS and OGCAS Scale: online game addicts (OGCAS Scale scores ≥32, and CIAS scores ≥5) and non-OGA Internet users (OGCAS Scale <32, and CIAS scores <5).

#### Stressful Life Events

The Chinese College-student Stress Questionnaire (CSSQ; Chi and Lin, 2005) is a 19-item self-report scale that assesses the frequency and intensity of the stressful life events for participants. The instrument includes five subscales: academic stress, social communication stress, job-related stress, daily hassles, and major life events, typified by phrases such as “I fail a mid-term exam or a final exam,” “I have a conflict with my best friend,” “I fail an important interview,” “I feel appetite loss and sleep disturbances,” “I have an experience of my family member’ serious illness and death.” Participants responded yes (score = 1) or no (score = 0) to items asking whether each stressful life event had happened to them within the past 6 months. For each “yes” response, participants reported the severity of the stressor by applying a score of 1 (no effect), 2 (mild effect), 3 (rather serious), or 4 (very serious). In the current sample, the internal consistency of the CCSQ (Cronbach’s α = 0.83) and the five subscales (academic stress, Cronbach’s α = 0.71; social communication stress, Cronbach’s α = 0.60; job-related stress, Cronbach’s α = 0.70; daily hassles, Cronbach’s α = 0.62; major life events, Cronbach’s α = 0.63) was acceptable.

#### Coping Styles

The Coping Style Questionnaire (CSQ; Roger et al., 1993) which consists of 62 items and six subscales (problem-solving, self-blame, help-seeking, fantasy, tolerance, and rationalization) was used to assess an individual’s coping ways with stress. The Chinese version of the CSQ (Xiao and Xu, 1996) was administered to the participants in the present study. ACSs include self-blame, fantasy, tolerance and rationalization, whereas confrontative coping strategies include problem-solving and help-seeking. Participants were required to provide a yes (score = 1) or no (score = 0) response to each item with higher scores reflecting a greater reliance on the associated coping strategy. In the current sample, the internal consistency of the CSQ (Cronbach’s α = 0.78) was sufficient. The Cronbach’s α for six subscales ranged from 0.50 to 0.67.

#### Personality Traits

The Revised short-form Eysenck Personality Questionnaire Short Scale (EPQ-RS; Barrett et al., 1998) which consists of a 48-item self-report scale with four subscales: psychoticism, extraversion, neuroticism, and social desirability has been widely used for measuring personality traits. We employed the EPQ-RS in Chinese (Qian et al., 2000) in the present study. These four traits have been shown to have good factorial similarity across 34 countries in a study that analyzed gender-specific data (Barrett et al., 1998). Participants provided a yes or no response, and responses were scored 1 or 0, respectively, except for some reverse-scored items. In the current sample, Cronbach’s α coefficient for the EPQ-RSC ranged from 0.62 to 0.72.

### Testing for Moderated Mediation

Simple mediation is said to occur when the causal effect of an independent variable on a dependent variable is transmitted by a mediator (Baron and Kenny, 1986). Moderated mediation occurs when mediation relations are contingent upon the level of a moderator, that is when the magnitude of a mediation effect is dependent upon a modulator (James and Brett, 1984; Muller et al., 2005). If the moderator (neuroticism in our study) differs across individuals, then moderated mediation indicates that the mediating process that intervenes between the casual factor and the outcome differs between people who differ in the moderator.

Analysis of Structural Equation Model was used to explore the interactive effects of stressful life events, ACSs and neuroticism on OGA. Stressful life events were measured using the total scores of CCSQ. OGA was measured using the total scores of OGCAS. ACSs were measured using the sum of four subscale scores: self-blame, fantasy, tolerance and rationalization. Neuroticism was measured using the subscale of Neuroticism in EPQ-RSC.

The model for this hypothesis consisted of stressful life events as an independent variable, neuroticism as a continuous moderator, ACSs as a mediator, OGA as a dependent variable, and gender and college year as control variables. According to the moderated mediation analysis steps developed by Muller et al. (2005), in equation 1, we estimated the effect of stressful life events (independent variable), neuroticism (moderator), stressful life events^∗^neuroticism (interaction1) on OGA (dependent variable). In equation 2, we estimated the effect of stressful life events, neuroticism, stressful life events^∗^neuroticism on ACSs (mediator). In equation 3, we estimated the effect of stressful life events, neuroticism, ACSs, ACSs^∗^neuroticism (interaction2), stressful life events^∗^neuroticism on OGA. As recommended by Frazier et al. (2004), all predictors were centered.

$$\begin{array}{c} Equation\;1:\;Online\;game\;addiction\;=\;c_{1}\;Stressful\;life\;events\;+\\ c_{2}\;Neuroticism\;+\;c_{3}\;stressful\;life\;events^{*}neuroticism\;+\;e_{1}\\ {Equation\text{ 2: }ACSs\text{ = a}}_{\text{1}}{Stressful\;life\;events\text{ + a}}_{2}\;Neuroticism\;\\ +\;a_{3}\;Stressful\;life\;events\text{*neuroticism}\;\text{+}\;{\text{e}}_{\text{2}}\\ Equation\;3:\;Online\;game\;addiction\;=\;c^{\prime }Stressful\;life\;events\;+\\ c^{\prime }{}_{2}\;Neuroticism\;\text{+}\;{\text{b}}_{\text{1}}\text{ACSs}\;\text{+}\;{\text{b}}_{\text{2}}\text{ACSs*}Neuroticism\;\text{+}\\ c^{\prime }{}_{3}\;Stressful\;life\;events*Neuroticism\;+\;e_{3} \end{array}$$

The indirect effect (moderated mediation) is calculated as (a_1_ + a_3_ Neuroticism) × (b_1_ + b_2_ Neuroticism).

### Data Analysis

Statistical analysis was performed in the Statistical Package for Social Sciences (SPSS, version 20.0 for Windows; Chicago, IL, USA) and Mplus (version 5, Muthén and Muthén, 2007). Descriptive analyses were performed on all variables. Baseline characteristics were compared between groups using the independent-sample *t*-test for quantitative variables and the chi-squared test for qualitative variables. Online game addict and non-addict groups were compared with independent-sample *t*-tests. Pearson correlation coefficients were used to assess the strengths of linear relationships between pairs of variables of interest. *P*-values are corrected by Bonferroni method for multiple comparisons and multiple correlations.

Moderated mediation models were estimated based on data from all 654 participants with valid measures of stressful life events, neuroticism, ACSs and OGA, controlling for relevant variables (i.e., gender and college year). Missing data are handled with full information maximum likelihood (Enders, 2001). The level of significance was set at *p* ≤ 0.05. For the hypothesized models, we evaluated model fit by using the comparative fit index (CFI), Tucker-Lewis fit index (TLI), root-mean-square error of approximation (RMSEA), standardized root-mean-square residual (SRMR), and Bayesian information criterion (BIC) as suggested by Chen (2007). A good model fit is indicated by a CFI larger than 0.95, a TLI larger than 0.95 and an RMSEA smaller than 0.05 (Kline, 2010).

## Results

### Sample

Of the 654 participants included in this study, 31 (4.7%) were identified as online game addicts (OGCAS: 44.97, 8.14; CIAS: 5.38, 1.78), and 623 (95.3%) were non-addicts (OGCAS: 19.38, 4.78; CIAS: 1.90, 1.37). Of the 31 addicts, 21 (77.4%) were males, leading to a gender difference between addicts and non-addicts (χ^2^ = 18.21, *p* < 0.001). Among non-addicts, 277 (44.5%) were males and 346 (55.5%) were females. There were no significant differences between the OGA group and the non-OGA group in terms of age, year in college, or major.

### Intergroup Differences in Questionnaire Outcomes

Questionnaire scores are presented in **Table 2**. In terms of stress, assessed with the CSSQ, the two groups’ scores were similar on the academic stress, job stress, daily hassles, interpersonal conflicts, and major events. Briefly, the CSQ revealed that the OGA group showed a greater propensity toward ACSs than the non-OGA group (self-blame: *t* = -3.81, *p* < 0.001; fantasy: *t* = -3.28, *p* = 0.001). Additionally, the OGA group’s scores were significantly higher on the neuroticism (*t* = -3.90, *p* < 0.001) subscales of the EPQ-RSC than those of the non-OGA group.

**Table 2:** Means and standard of stressful life events, coping strategies, personality traits, and online game addiction (OGA).

Variables	Addicts (*N* = 31)		Non-addicts (*N* = 623)		*t*
	*M*	*SD*	*M*	*SD*	
Total CSSQ scores	24.48	14.19	21.90	11.14	-1.19
Academic stress	7.38	4.69	5.82	3.55	-2.25
Job stress	5.31	3.56	5.02	3.09	-0.49
Daily hassles	5.89	3.07	5.09	3.05	-1.37
Interpersonal conflicts	3.93	3.87	3.94	2.99	0.02
Major events	1.97	2.49	2.03	2.20	0.14
CSQ					
Problem-solving	7.68	2.66	8.97	2.31	2.25
Help-seeking	5.00	2.39	5.59	2.32	1.23
fantasy	5.44	2.38	4.15	2.06	-3.03^∗∗^
Rationalization	5.04	2.13	4.21	1.95	-2.24
Self blame	4.72	2.42	3.38	2.07	-3.15^∗∗^
Withdrawal	5.40	2.00	4.57	1.95	-2.07
EPQ-RSC					
Neuroticism	6.53	2.11	4.53	2.77	-3.90^∗∗∗^
Psychoticism	7.53	1.57	6.71	1.55	-2.80
Extraversion	7.03	2.33	7.51	2.54	1.02
Social desirability	6.47	1.87	6.39	2.05	-0.19
OGCAS scores	44.97	8.14	19.38	4.78	-27.49^∗∗∗^
CIAS scores	5.96	0.97	1.91	1.24	-16.66^∗∗∗^

**P*-values are corrected by Bonferroni method for multiple comparisons. ^∗∗^*p* < 0.005, ^∗∗∗^*p* < 0.001. CCSQ, The Chinese College-student Stress Questionnaire; CSQ, Coping Style Questionnaire; EPQ-RSC, the Revised Eysenck Personality Questionnaire Short Scale for Chinese; OGCAS, Online Game Cognitive Addiction Scale; CIAS, Chinese version of the Internet Addiction Scale.*

### Relationships between Stressful Life Events, ACSs, Neuroticism, and OGA

Correlations between variables of interest are presented separately for males and females in **Table 3**. Significant gender differences were observed, with only males showing statistically significant correlations among stressful life events, academic stress, ACSs, psychoticism and the OGCAS scores. Specifically, in males, ACSs correlated with stressful life events (*r* = 0.33, *p* < 0.001), academic stress(*r* = 0.23, *p* < 0.001), psychoticism (*r* = 0.34, *p* < 0.001) and the OGCAS scores (*r* = 0.25, *p* < 0.001).

**Table 3:** Correlations between stressful life events, coping strategies, personality traits, and OGA for males (*N* = 298) and females (*N* = 356).

Variables	1	2	3	4	5	6
(1) Avoidant coping styles	-	0.25^∗∗∗^	0.07	0.27^∗∗∗^	0.24^∗∗∗^	0.10
(2) Psychoticism	0.34^∗∗∗^	-	0.13	0.22^∗∗∗^	0.22^∗∗∗^	0.08
(3) Neuroticism	0.07	0.08	-	0.07	0.02	-0.05
(4) Stressful life events	0.33^∗∗∗^	0.20^∗∗^	0.01	-	0.74^∗∗∗^	0.06
(5) Academic stress	0.23^∗∗∗^	0.18^∗∗^	-0.003	0.69^∗∗^	-	0.07
(6) OGA	0.25^∗∗∗^	0.09	-0.07	0.13	0.09	-

*Correlations for males are below the diagonal and for females are above the diagonal. *P*-values are corrected by Bonferroni method for multiple correlations. ^∗∗^*p* < 0.005, ^∗∗∗^*p* < 0.001. Avoidant coping styles, sum of four subscale scores of CSQ: self blame, fantasy, withdrawal and rationalization; Psychoticism, the subscale scores of psychoticism in EPQ-RSC; Neuroticism, the subscale scores of psychoticism in EPQ-RSC; Stressful life events, total scores of CSSQ; Academic stress, the subscale scores of academic stress in CSSQ; OGA, total scores of OGCAS.*

In females, ACSs correlated with stressful life events (*r* = 0.27, *p* < 0.001), academic stress (*r* = 0.24, *p* < 0.001), and psychoticism (*r* = 0.25, *p* < 0.001). There was no significant correlation between ACSs and the OGCAS scores (**Table 3**).

### Moderated Mediation

The measurement model was first tested for an acceptable fit to the data through a CFA, and it had good fit to the data, CFI = 0.95, RMSEA = 0.052 [90% CI = 0.042–0.061].

**FIGURE 2:**
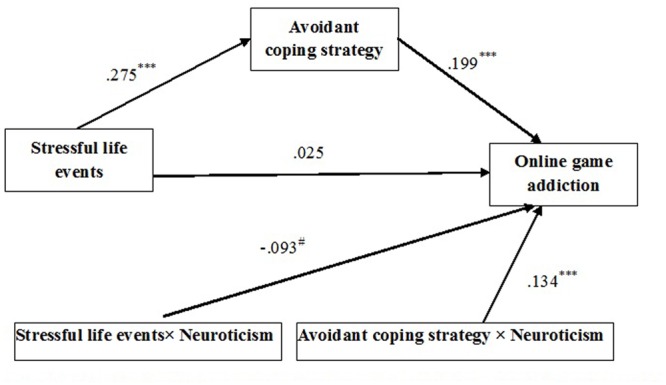
**Results of mediation analysis for online game addiction**. Values presented are standardized coefficients (^∗∗∗^*P* < 0.00; ^#^*P* at a marginally significant level).

After an acceptable measurement model was developed, the structural model was tested. **Figure 2** depict the results of Structural equation modeling (SEM) analyses. The first stage was to establish a simple mediation model which OGA was regression on stress mediated by ACSs. The simple mediation model displayed satisfactory model fit indices (χ2 = 2.48, df = 3, *p* < 0.001, CFI = 1.00, TLI = 1.00, RESEA < 0.001, SRMR = 0.01, BIC = 6714.64). The standardized path coefficients from stressful life events to ACSs [β = 0.277 (*SE* = 0.043), *p* < 0.001] and from ACSs to OGA [β = 0.195 (*SE* = 0.045), *p* < 0.001] were significant. The path from stressful life events to OGA was also significant [β = 0.09 (*SE* = 0.05), *p* < 0.05].

The second stage was to test the moderated mediation model. The hypothesized model obtained acceptable model fit indices (χ^2^ = 1.78, df = 1, *p* < 0.001, CFI = 0.99, TLI = 0.91, RESEA = 0.036, SRMR = 0.01, BIC = 9957.67). Analysis of mediation indicated that the standardized path coefficients from stressful life events to ACSs [β = 0.275 (*SE* = 0.039), *p* < 0.001] and from ACSs to OGA [β = 0.199 (*SE* = 0.046), *p* < 0.001] were significant. The path from stressful life events to OGA was non-significant [β = 0.025 (*SE* = 0.044), *p* > 0.05]. As hypothesized, ACSs fully mediated the positive relationship between stressful life events and OGA. Moreover, the interaction effect of neuroticism on stress life evens toward OGA was at a marginally significant level [β = -0.093 (*SE* = 0.049), *p* = 0.06]. Importantly, the interaction effect of neuroticism on ACS toward OGA was positive and statistically significant [β = 0.134 (*SE* = 0.051), *p* < 0.001] (**Figure 2**). The regression coefficients of three models are presented in **Table 4**. The significant interactions observed were indicative of moderated mediation, meaning that ACS (the mediating process) that intervened between stress (the independent variable) and OGA (the dependent variable) was different for people who differ with respect to neuroticism (the moderator).

**Table 4:** Model for the moderated-mediation hypothesis.

Predictors	Outcomes								
	OGA (Model 1)			ACSs (Model 2)			OGA (Model 3)		
	*b*	*SE*	*p*	*b*	*SE*	*p*	*b*	*SE*	*p*
Stressful life events	0.09	0.04	0.03						
Neuroticism	-0.02	0.05	0.73						
Stressful life events^∗^neuroticism	-0.04	0.05	0.46						
Stressful life events				0.28	0.04	< 0.001			
Neuroticism				0.04	0.04	0.30			
Stressful life events^∗^neuroticism				0.03	0.06	0.67			
Stressful life events							0.03	0.04	0.58
Neuroticism							-0.03	0.04	0.50
ACSs							0.20	0.05	< 0.001
ACSs^∗^Neuroticism							0.13	0.05	0.008
Stressful life events^∗^Neuroticism							-0.09	0.05	0.06

*ACSs, avoidant coping styles, sum of four subscale scores of CSQ: self blame, fantasy, withdrawal and rationalization; Psychoticism, the subscale scores of psychoticism in EPQ-RSC; Neuroticism, the subscale scores of psychoticism in EPQ-RSC; Stressful life events, total scores of CSSQ; OGA, total scores of OGCAS.*

## Discussion

This study focused on exploring how stress and individual psychological characteristics, such as ACSs and neuroticism, relate to OGA in Chinese college students. The findings provide empirical evidence indicating that stressful life events may be an important predictor of OGA. ACSs and neuroticism correlated with OGA, especially for males. The relationship between stressful life events and OGA was fully mediated by ACSs. Importantly, the magnitude of this mediation effect was dependent upon a moderator, namely neuroticism.

In the current sample, 4.7% of the college-student participants and 7.05% of the male students were classified as online game addicts because they fulfilled OGCAS and IAS criteria (see Materials and Methods). This incidence rate was lower than rates in the general population and Chinese college students reported in previous studies. A survey from China Internet Network Information Center (2009) showed that incidence rate in 13–24-year-olds was 9.72%. Luo et al. (2010) reported a 12.2% incidence of Internet addiction disorder among college students using the Chinese Internet Addiction Scale (CIAS, Ko et al., 2009a), and Yu (2010) reported a 17% incidence of OGA among Chinese college students using the OGA scale (OGAS, Huang, 2004). Because our study is 6 and 12 years more recent than these studies, respectively, these data may reflect a decreasing prevalence of OGA among Chinese college students. However, these results should be interpreted with caution because different measures were used, and our study had stricter diagnostic criteria than were employed in the prior studies. If OGA is truly declining, it might be due to prevention programs, including stress management, emotional regulation skill training, and coping skill training, which were devised to moderate the influence of excessive online gaming among college students on university campuses. Furthermore, our previous works found that prevalence of GPIU was 13.2% in this study population (Li et al., 2009), suggesting that college student Internet users might be at greater risk of developing GPIU than OGA.

It is interesting that the current study revealed a twofold greater OGA in males than in females. However, there were no significant gender differences found in prevalence of GPIU in this study population (Li et al., 2009). These findings are supported by previous studies which have shown significant distinction in gender differences between GPIU and OGA. Williams et al. (2009) reported that female video game players comprised 40% all players, and females over 18 comprised more of the game-playing population than did males under 17. However, males might be more susceptible to excessive playing, especially in Massive Multiplayer Online Games (Hou, 2008; Wang and Wang, 2008). For SPIU, male addicts were more likely to engage in online game playing, gambling and sex (Wong et al., 2013; Dufour et al., 2016), while female addicts were more likely to engage in online shopping and social networking (Seock and Bailey, 2008; Pujazonzazik and Park, 2010). Online gaming is not only a youth-culture phenomenon, but is also a component of college social life. As such, males and females may experience different levels of motivation for and enjoyment of game playing. For example, male players were shown to be more achievement-oriented and more willing to maintain relationships with other players than female players (Ko et al., 2005; Dmitri et al., 2009). In addition, males played more highly aggressive and addictive games, such as combat or adventure games (Anderson and Dill, 2000), felt more anxiety when they did not advance in the games, and invested more time to conquer them. In contrast, female players reported engaging in significantly more exercise than male players, and they showed a lower body mass index (BMI) than male players and the National female mean, suggesting that female players were healthier than male players as well as females in the general population. Within romantic relationships where both partners played, female players reported higher general happiness than their male counterparts (Dmitri et al., 2009), which may indicate that they had less cognitive distortion related to online gaming (Li and Wang, 2013). Gender differences in OGA suggest that males may be more susceptible to online game overuse and addiction than females.

Major sources of stress among college students include academic, interpersonal, and job-related stressors (Brougham et al., 2009). Our results indicated that online game addicts used more ACSs (self-blame, fantasy, withdrawal, and rationalization) than non-addicts when facing a stressful event. These finding are consistent with our previous work which has shown that GPIU addicts have confronted with more stressful life events and used more ACSs than non-addicts (Li et al., 2009), suggesting that stress and ACSs might be significant pre-existing risk factors in either GPIU or OGA. Moreover, especially in males, scores on ACSs correlated positively the CCSQ and OGCAS scores. One possible interpretation is that individuals who use ACSs rather than problem-solving are more likely to play online games to cope with stress. The improved self-esteem and satisfaction derived from good game skills may hinder players from reducing their online game play (Smyth, 2007). However, the more individuals indulged in online game playing, the more stressful life events may occur, and the more ACSs they will use, resulting in a positive feedback loop. Interestingly, our results also showed that the relationships among stressful life events, ACSs, and OGA for males were much stronger than those for females. These differences may be relevant for explaining gender difference in online game overuse among college students.

The results of SEM analyses showed that ACSs positively and fully mediate the effects of stressful life events on OGA. Given that maladaptive coping was a potential mediator between stress (e.g., family stress, childhood trauma, and daily hassles) and its psychological and behavioral outcomes including depression, PTSD, and internet addiction (Choi et al., 2015; Ye and Zheng, 2016), it is not strange that we found that OGA were affected by stressful life events indirectly through ACSs (e.g., self-blame, fantasy, withdrawal or rationalization) rather than stressful life events directly.

Further, a stress × neuroticism marginally significant interaction observed suggested that neuroticism directly moderates the effect of stressful life events on OGA, so that in emotionally stable individuals an increase in stressful life events is unnecessarily related to a increase in OGA, while in individuals high in neuroticism it is related to an increase of OGA.

Interestingly, the magnitude of the ACS mediation effect was dependent upon neuroticism, suggesting that individuals with impulse control and emotional instability issues are more likely to use ACSs and are more susceptible to OGA when they encounter stress. Previous studies showed neuroticism to be positively related to Internet addiction, suggesting that individuals with emotional instability issues may be more susceptible to problematic Internet use (Yao et al., 2014). Highly neurotic women have been reported to use online social networking platforms with elevated frequency, likely to reduce social loneliness (Hamburger and Ben-Artzi, 2000). Furthermore, neurotic individuals may find social interactions to be more rewarding online than in real life if they experience difficulties in real social interactions (Amichai-Hamburger et al., 2002). Thus, neurotic individuals may be more likely to spend time playing online games, even becoming compulsive players, and may be more likely to experience withdrawal when they stop playing (Anolli et al., 2005).

### Limitation

The present study had several limitations. First, the cross-sectional, correlative design does not allow conclusions to be made about the causal relationships among stress, ACSs, and neuroticism on OGA. In the future, a longitudinal study with individual interviews would be needed to better elucidate the influence of stress on OGA among college students. Second, even though there is similar diversity in students’ places of origin, major and cultural circumstances in the three universities, the possible cluster effect by university should be taken into account. Third, this sample was relatively small and not fully representative of Chinese college students. Therefore, its generalizability is limited. A larger representative sample should be studied to confirm the present results.

## Conclusion

The present results indicated that neuroticism acts as a moderator, which interacts with the most robust predictor, ACSs, to influence OGA. The observed ACS × neuroticism interaction effect on OGA has clear clinical relevance. Compared to healthy controls, neurotic individuals appear to have difficulty dealing with stress, leading to more complaints, shyness, and tendencies to make avoidant, dysfunctional coping choices, rather than problem-solving (Gunthert et al., 1999). From these findings, it is reasonable to speculate that neurotics who used online games as a means of escaping from their real-life difficulties are more susceptible to OGA. Future intervention programs should take into account cognitive styles, particularly of neurotic individuals, and employ coping strategy training.

## Author Contributions

HL: Research design, data analysis and manuscript preparation. JW: Sampling and data collection. XY: Sampling. YZ: Data analysis.

## Conflict of Interest Statement

The authors declare that the research was conducted in the absence of any commercial or financial relationships that could be construed as a potential conflict of interest.

## References

[B1] Amichai-Hamburger Y. Wainapel G. Fox S. (2002). “On the internet no one knows i’m an introvert”: extroversion, neuroticism, and internet interaction. *Cyberpsychol. Behav.* 5 125–128. 10.1089/109493102753770507 12025878

[B2] Anderson C. Dill K. (2000). Video games and aggressive thoughts, feelings, and behaviorin the laboratory and in life. *J. Pers. Soc. Psychol.* 78 772–790. 10.1037/0022-3514.78.4.772 10794380

[B3] Anolli L. Villani D. Riva G. (2005). Personality of people using chat: an on–line research. *Cyberpsychol. Behav.* 8 89–95. 10.1089/cpb.2005.8.89 15738696

[B4] Baron R. M. Kenny D. A. (1986). The moderator–mediator variable distinction in social psychological research: conceptual, strategic, and statistical considerations. *J. Pers. Soc. Psychol.* 51 1173–1182. 10.1037/0022-3514.51.6.1173 3806354

[B5] Barrett P. T. Petrides K. V. Eysenck S. B. G. Eysenck H. J. (1998). The Eysenck personality questionnaire: an examination of the factorial similarity of P, E, N, and l across 34 countries. *Pers. Individ. Dif.* 25 805–819. 10.1016/S0191-8869(98)00026-9

[B6] Billieux J. Chanal J. Khazaal Y. Rochat L. Gay P. Zullino D. (2011). Psychological predictors of problematic involvement in massively multiplayer online role–playing games: illustration in a sample of male cybercafé players. *Psychopathology* 44 165–171. 10.1159/000322525 21372629

[B7] Block J. J. (2008). Issues for DSM-V: internet addiction. *Am. J. Psychiatry* 165 306–307. 10.1176/appi.ajp.2007.07101556 18316427

[B8] Brougham R. R. Zail C. M. Mendoza C. M. Miller J. R. (2009). Stress, sex differences, and coping strategies among college students. *Curr. Psychol.* 28 85–97. 10.1007/s12144-009-9047-0

[B9] Burgess S. R. Stermer S. P. Burgess M. C. R. (2012). Video game playing and academic performance in college students. *Coll. Stud. J.* 46 376–387.

[B10] Cao F. Su L. (2007). Internet addiction among Chinese adolescents: prevalence and psychological features. *Child Care Health Dev.* 33 275–281. 10.1111/j.1365-2214.2006.00715.x 17439441

[B11] Charlton J. P. Ian D. W. (2010). Validating the distinction between computer addiction and engagement: online game playing and personality. *Behav. Inf. Technol.* 29 601–613. 10.1080/01449290903401978

[B12] Chen F. F. (2007). Sensitivity of goodness of fit indexes to lack of measurement invariance. *Struct. Equ. Modeling* 14 464–504. 10.1080/10705510701301834

[B13] Cheng C. Sun P. Mak K. K. (2015). Internet addiction and psychosocial maladjustment: avoidant coping and coping inflexibility as psychological mechanisms. *Cyberpsychol. Behav. Soc. Netw.* 18 539–546. 10.1089/cyber.2015.0121 26348815

[B14] Chi S. Lin W. J. (2005). Stressful events, personality, social support and mood states of college seniors (in Chinese). *Chin. J. Ment. Health* 19 513–516.

[B15] China Internet Network Information Center (2009). *It is Critical Time to Protect Adolescents and Young Adults from Internet Overuse.* Available at: http://www.cnnic.cn/hlwfzyj/hlwfzzx/qsnwm/201206/t20120612_26783.htm

[B16] China Online Game Users Survey Report (2008). *iResearch Consulting Group, Shanghai*. Available at: http://www.cnnic.cn/wapweb/sjbg/201611/t20161109_55931.htm

[B17] Choi K. W. Sikkema K. J. Velloza J. Marais A. Jose C. Stein D. J. (2015). Maladaptive coping mediates the influence of childhood trauma on depression and ptsd among pregnant women in south africa. *Arch. Womens Ment. Health* 18 731–738. 10.1007/s00737-015-0501-8 25578632 PMC4500677

[B18] Collins E. Freeman J. Chamarro–Premuzic T. (2012). Personality traits associated with problematic and non–problematic massively multiplayer online role playing game use. *Pers. Individ. Dif.* 52 133–138. 10.1016/j.paid.2011.09.015

[B19] Connor-Smith J. K. Flachsbart C. (2007). Relations between personality and coping: a meta–analysis. *J. Pers. Soc. Psychol.* 93 1080–1107. 10.1037/0022-3514.93.6.1080 18072856

[B20] Davis R. A. (2001). A cognitive–behavioral model of pathological Internet use. *Comput. Hum. Behav.* 17 187–195. 10.1016/S0747-5632(00)00041-8

[B21] Dickey M. D. (2007). Game design and learning: a conjectural analysis of how massively multiple online role–playing games (mmorpgs) foster intrinsic motivation. *Educ. Technol. Res. Dev.* 55 253–273. 10.1007/s11423-006-9004-7

[B22] Dmitri W. Mia C. Scott C. Nick Y. (2009). Looking for gender: gender roles and behaviors among online gamers. *J. Commun.* 59 700–725. 10.1111/j.1460-2466.2009.01453.x

[B23] Douglas A. C. Mills J. E. Niang M. Stepchenkova S. Byun S. Ruffinia C. (2008). Internet addiction: meta-synthesis of qualitative research for the decade 1996–2006. *Comput. Hum. Behav.* 24 3027–3044. 10.1016/j.chb.2008.05.009

[B24] Dufour M. Brunelle N. Tremblay J. Leclerc D. Cousineau M. M. Khazaal Y. (2016). Gender difference in internet use and internet problems among quebec high school students. *Can. J. Psychiatry* 61 663–668. 10.1177/0706743716640755 27310231 PMC5348090

[B25] Enders C. K. (2001). The performance of the full information maximum likelihood estimator in multiple regression models with missing data. *Educ. Psychol. Meas.* 61 713–740. 10.1177/0013164401615001

[B26] Frazier P. A. Tix A. P. Barron K. E. (2004). Testing moderator and mediator effects in counseling psychology research. *J. Couns. Psychol.* 51 115–134. 10.1037/0022-0167.51.1.115

[B27] Grusser S. M. Morsen C. P. Wolfling K. Flor H. (2007). The relationship of stress, coping, effect expectancies and craving. *Eur. Addict. Res.* 13 31–38. 10.1159/000095813 17172777

[B28] Gunthert K. C. Cohen L. H. Armeli S. (1999). The role of neuroticism in daily stress and coping. *J. Pers. Soc. Psychol.* 77 1087–1100. 10.1037/0022-3514.77.5.1087 10573882

[B29] Hamburger Y. A. Ben-Artzi E. (2000). The relationship between extraversion and neuroticism and the different uses of the internet. *Comput. Hum. Behav.* 16 441–449. 10.1016/S0747-5632(00)00017-0

[B30] Hills H. Norvell N. (1991). An examination of hardiness and neuroticism as potential moderators of stress outcomes. *Behav. Med.* 17 31–38. 10.1080/08964289.1991.9937550 2036495

[B31] Hosoi K. (2005). “Possibilities and prospects of on–line games in Asia,” in *Gaming, Simulations, and Society; Research Scope and Perspective* eds Shiratori R. Kato F. Arai K. (Berlin: Springer) 269–277.

[B32] Hou C. I. (2008). A cross–cultural comparison of gender representation in massively multiplayer online role–playing games: a study of taiwan and the united states. *China Media Res.* 4 13–25.

[B33] Huang Y. H. (2004). *Real Life in Virtual World: Online Game Addiction and its Related Factors*. (unpublished master’s thesis). Institute of Communication, Shih Hsih University Taipei.

[B34] James L. R. Brett J. M. (1984). Mediators, moderators, and test for mediation. *J. Appl. Psychol.* 69 307–321.

[B35] Jiang C. Yang L. G. Gao Q. Chen G. W. Shen J. L. (2007). The current situation of University students network games and causal analysis(In Chinese). *Health Med. Res. Pract.* 4 69–71.

[B36] Karakus T. Inal Y. Cagiltay K. (2008). A descriptive study of turkish high school students’ game–playing characteristics and their considerations concerning the effects of games. *Comput. Hum. Behav.* 24 2520–2529. 10.1016/j.chb.2008.03.011

[B37] Kardefelt-Winther D. (2014). Problematizing excessive online gaming and its psychological predictors. *Comput. Hum. Behav.* 31 118–122. 10.1016/j.chb.2013.10.017

[B38] Kim E. J. Namkoong K. Ku T. Kim S. J. (2008). The relationship between online game addiction and aggression, self–control and narcissistic personality traits. *Eur. Psychiatry* 23 212–218. 10.1016/j.eurpsy.2007.10.010 18166402

[B39] Kline R. B. (2010). *Principles and Practice of Structural Equation Modeling* 3rd Edn. New York, NY: Guilford Press.

[B40] Ko C. H. Yen J. Y. Chen C. C. Chen S. H. Yen C. F. (2005). Gender differences and related factors affecting online gaming addiction among taiwanese adolescents. *J. Nerv. Ment. Dis.* 193 273–277. 10.1097/01.nmd.0000158373.85150.57 15805824

[B41] Ko C. H. Yen J. Y. Chen S. H. Yang M. J. Lin H. C. Yen C. F. (2009a). Proposed diagnostic criteria and the screening and diagnosing tool of Internet addiction in college students. *Compr. Psychiatry* 50 378–384. 10.1016/j.comppsych.2007.05.019 19486737

[B42] Ko C. H. Yen J. Y. Liu S. C. Huang C. F. Yen C. F. (2009b). The associations between aggressive behaviors and internet addiction and online activities in adolescents. *J. Adolesc. Health* 44 598–605. 10.1016/j.jadohealth.2008.11.011 19465325

[B43] Kuss D. J. Griffiths M. D. Binder J. F. (2013a). Internet addiction in students: prevalence and risk factors. *Comput. Hum. Behav.* 29 959–966. 10.1016/j.chb.2013.04.002

[B44] Kuss D. J. Rooij A. J. V. Shorter G. W. Griffiths M. D. Mheen D. V. D. (2013b). Internet addiction in adolescents: prevalence and risk factors. *Comput. Hum. Behav.* 29 959–966. 10.1016/j.chb.2013.04.002

[B45] Laconi S. Tricard N. Chabrol H. (2015). Differences between specific and generalized problematic internet uses according to gender, age, time spent online and psychopathological symptoms. *Comput. Hum. Behav.* 48 236–244. 10.1016/j.chb.2015.02.006

[B46] Lafrenière M. A. K. Vallerand R. J. Donahue R. Lavigne G. L. (2009). On the costs and benefits of gaming: the role of passion. *CyberPsychol. Behav.* 12 285–290. 10.1089/cpb.2008.0234 19366320

[B47] Lai C. H. Lin C. Y. Chen C. H. Gwung H. L. Li C. H. (2013). “Can internet usage positively or negatively affect interpersonal relationship?,” in *Smart Innovation, Systems and Technologies* Vol. 20 eds Chang R. S. Jain L. C. Peng S. L. (Berlin: Springer) 373–382.

[B48] Lei L. Yang Y. Liu M. (2006). The relationship between adolescents’ neuroticism, internet service preference, and internet addiction. *Acta Psychol. Sin.* 38 375–381.

[B49] Lemmens J. S. Valkenburg P. M. Peter J. (2009). Development and validation of a game addiction scale for adolescents. *Media Psychol.* 12 77–95. 10.1371/journal.pone.0061098

[B50] Li H. Wang J. Wang L. (2008a). The difference of mental health levels and personality traits between Internet social addition and Internet game addition in college students (in Chinese). *Chin. J. Clin. Psychol.* 16 413–416.

[B51] Li H. Wang J. Wang L. (2009). A survey on the generalized problematic internet use in Chinese college students and its relations to stressful life events and coping style. *Int. J. Ment. Health Addict.* 7 333–346. 10.1007/s11469-008-9162-4

[B52] Li H. Wang L. Wang J. Q. (2008b). Development of Internet game cognition–addition Scale in college students of China (in Chinese). *Chin. J. Clin. Psychol.* 22 319–322.

[B53] Li H. Wang S. (2013). The role of cognitive distortion in online game addiction among Chinese adolescents. *Child. Youth Serv. Rev.* 35 1468–1475. 10.1016/j.childyouth.2013.05.021

[B54] Luo Z. H. Wan J. J. Liu Q. X. Fang X. Y. (2010). The relationship of internet Use, internet special self–efficacy and internet addiction in university students. *Psychol. Dev. Educ.* 6 618–626.

[B55] Mccusker C. G. Brown K. (1991). The cue–responsivity phenomenon in dependent drinkers: ‘personality’ vulnerability and anxiety as intervening variables. *Br. J. Addict.* 86 905–912. 10.1111/j.1360-0443.1991.tb01846.x 1912743

[B56] Mehroof M. Griffiths M. D. (2010). Online gaming addiction: the role of sensation seeking, self–control, neuroticism, aggression, state anxiety, and trait anxiety. *Cyberpsychol. Behav. Soc. Netw.* 13 313–316. 10.1089/cyber.2009.0229 20557251

[B57] Morin C. M. Rodrigue S. Ivers H. (2003). Role of stress, arousal, and coping skills in primary insomnia. *Psychosom. Med.* 65 259–267. 10.1097/01.PSY.0000030391.09558.A3 12651993

[B58] Muller D. Judd C. M. Yzerbyt V. Y. (2005). When moderation is mediated and mediation is moderated. *J. Pers. Soc. Psychol.* 89 852–863. 10.1037/0022-3514.89.6.852 16393020

[B59] Muthén L. Muthén B. (2007). *Mplus Users: Statistical Analysis with Latent Variables – User’s Guide*. Los Angeles, CA: Muthén & Muthén.

[B60] Pujazonzazik M. Park M. J. (2010). To tweet, or not to tweet: gender differences and potential positive and negative health outcomes of adolescents’ social internet use. *Am. J. Mens. Health* 4 77–85. 10.1177/1557988309360819 20164062

[B61] Qahri-Saremi H. Turel O. (2016). School engagement, information technology use, and educational development: an empirical investigation of adolescents. *Comput. Educ.* 102 65–78. 10.1016/j.compedu.2016.07.004

[B62] Qian M. Y. Wu G. C. Zhu R. C. Zhang S. (2000). Development of the revised Eysenck personality questionnaire short scale for Chinese (in Chinese). *Acta Psychol. Sin.* 32 317–323. 10.1017/dmp.2015.64

[B63] Rice L. Markey P. M. (2008). The role of extraversion and neuroticism in influencing anxiety following computer-mediated interactions. *Pers. Individ. Dif.* 46 35–39. 10.1016/j.paid.2008.08.022

[B64] Roger D. Jarvis G. Najarian B. (1993). Detachment and coping: the construction and validation of a new scale for measuring coping strategies. *Pers. Individ. Dif.* 15 619–626. 10.1016/0191-8869(93)90003-L

[B65] Ross S. E. Niebling L. C. Heckert T. M. (1999). Sources of stress among college students. *Coll. Stud. J.* 33 312–317.

[B66] Seock Y. K. Bailey L. R. (2008). The influence of college students’ shopping orientations and gender differences on online information searches and purchase behaviours. *Int. J. Consum. Stud.* 32 113–121.

[B67] Sigurdsso J. F. Gudjonsso G. H. (2009). Personality characteristics of drug–dependent offenders. *Nord. J. Psychiatry* 49 33–38. 10.3109/08039489509011881

[B68] Smyth J. M. (2007). Beyond self–selection in video game play: an experimental examination of the consequences of massively multiplayer online role–playing game play. *Cyberpsychol. Behav.* 10 717–721. 10.1089/cpb.2007.9963 17927543

[B69] Wang H. Y. Wang Y. S. (2008). Gender differences in the perception and acceptance of online games. *Br. J. Educ. Technol.* 39 787–806.

[B70] Wang Y. Gao W. B. (2008). A study of hear t rate variability frequency field characteristics of internet addicts (in Chinese). *Chin. J. Clin. Psychol.* 16 316–323.

[B71] Williams D. Consalvo M. Caplan S. Yee N. (2009). Looking for gender (LFG): gender roles and behaviors among online gamers. *J. Commun.* 59 700–725. 10.1111/j.1460-2466.2009.01453.x

[B72] Wong G. Zane N. Saw A. Chan A. K. K. (2013). Examining gender differences for gambling engagement and gambling problems among emerging adults. *J. Gambl. Behav.* 29 171–189. 10.1007/s10899-012-9305-1 PMC4736715 22585283

[B73] Xiao J. H. Xu X. F. (1996). Reliability and validity of coping style questionnaire(in Chinese). *Chin. J. Ment. Health* 10 164–168.

[B74] Yan W. S. Li Y. H. Sui N. (2014). The relationship between recent stressful life events, personality traits, perceived family functioning and internet addiction among college students. *Stress Health* 30 3–11. 10.1002/smi.2490 23616371

[B75] Yang C. K. Choe B. M. Baity M. Lee J. H. Cho J. S. (2005). Scl–90–r and 16pf profiles of senior high school students with excessive internet use. *Can. J. Psychiatry* 50 407–414. 16086538 10.1177/070674370505000704

[B76] Yao M. Z. He J. Ko D. M. Pang K. (2014). The influence of personality, parental behaviors, and self–esteem on internet addiction: a study of Chinese college students. *Cyberpsychol. Behav. Soc. Netw.* 17 104–110. 10.1089/cyber.2012.0710 24003966 PMC3924803

[B77] Ye B. Zheng Q. (2016). The effects of stress on college students’ internet addiction. *J. Psychol. Sci.* 39 621–627.

[B78] Young K. S. (1998). Internet addiction: the emergence of a new clinical disorder. *CyberPsychol. Behav.* 1 237–244. 10.1007/s10899-011-9287-4

[B79] Yu L. (2013). Neuroticism as a moderator of the effects on adolescent stress and depressive symptoms: a longitudinal study. *Stud. Psychol. Behav.* 11 411–416.

[B80] Yu Q. (2010). An investigation and analysis on online game addiction of college students. *J. Panzhihua Univ.* 27 112–115.

[B81] Zhou Y. Li Z. (2009). Online game addiction among Chinese college students measurement and attribution. *Stud. Health Technol. Inf.* 144 149–154. 19592753

[B82] Zhu K. J. Wu H. R. (2004). Psychosocial factors of Internet addiction disorder in college students (in Chinese). *Chin. J. Ment. Health* 18 796–798.

